# A Role of the ABCC4 Gene Polymorphism in Airway Inflammation of Asthmatics

**DOI:** 10.1155/2017/3549375

**Published:** 2017-06-04

**Authors:** Sailesh Palikhe, Udval Uuganbayar, Hoang Kim Tu Trinh, Ga-Young Ban, Eun-Mi Yang, Hae-Sim Park, Seung-Hyun Kim

**Affiliations:** ^1^Department of Allergy and Clinical Immunology, Ajou University School of Medicine, Suwon, Republic of Korea; ^2^Department of Biomedical Sciences, Graduate School of Ajou University, Suwon, Republic of Korea; ^3^Clinical Trial Center, Ajou University Medical Center, Suwon, Republic of Korea

## Abstract

The ATP-binding cassette subfamily C member 4 gene encodes a transmembrane protein involved in the export of proinflammatory molecules, including leukotriene, prostaglandin, and sphingosine-1-phosphate across the plasma membrane. Those metabolites play important roles in asthma. We investigated the potential associations between *ABCC4* gene polymorphisms and asthma phenotype. In total, 270 asthma patients and 120 normal healthy controls were enrolled for a genetic association study. Two polymorphisms (−1508A>G and −642C>G) in the *ABCC4* promoter were genotyped. The functional variability of the promoter polymorphisms was analyzed by luciferase reporter assay. Inflammatory cytokine levels were measured by enzyme-linked immunosorbent assay. Serum and urinary eicosanoid metabolites, sphingosine-1-phosphate, were evaluated by quadrupole time-of-flight mass spectrometry. Asthma patients carrying the G allele at −1508A>G had significantly higher serum levels of periostin, myeloperoxidase, and urinary levels of 15-hydroxyeicosatetraenoic acid and sphingosine-1-phosphate (*P* = 0.016, *P* = 0.027, *P* = 0.032, and *P* = 0.010, resp.) compared with noncarrier asthma patients. Luciferase activity was significantly enhanced in human epithelial A549 cells harboring a construct containing the −1508G allele (*P* < 0.01 for each) compared with a construct containing the −1508A allele. A functional polymorphism in the *ABCC4* promoter, −1508A>G, may increase extracellular 15-hydroxyeicosatetraenoic acid, sphingosine-1-phosphate, and periostin levels, contributing to airway inflammation in asthmatics.

## 1. Introduction

Multidrug resistance protein 4 (ABCC4) is a member of the family of ATP-binding cassette transporters required for the active transport of many bioactive substrates across the cell membrane [1]. ABCC4 pumps various substrates, including eicosanoids, cyclic nucleotides, bile salts, steroids, and other drugs, out of the cell to control multiple cellular signaling processes, including inflammation, cancer, cardiovascular homeostasis, platelet function, endothelial barrier function, vascular smooth muscle cell proliferation, and vasodilation [1–6].

Eicosanoids such as prostaglandin E2 (PGE_2_) and cysteinyl leukotriene (LT) E4, along with cyclic adenosine monophosphate (cAMP), are exported via ABCC4, and these molecules play important roles in airway inflammation [7, 8]. Previous studies have demonstrated that intracellular elevation of cAMP exerts an anti-inflammatory effect and PGE_2_ induces cAMP [9, 10]. The exposure of eosinophils to LTE4 also induces cAMP production [11]. Additionally, cAMP is involved in the induction and regulation of T helper (Th2) immunity, particularly in allergic asthma via dendritic cells [12]. Pharmacological inhibition of ABCC4 reduces the migration of human dendritic cells, indicating an important role for ABCC4 in human immunology [13]. Therefore, ABCC4 may play an important role in inflammatory diseases, particularly in asthma, by regulating the intracellular concentration of cAMP [12]. In addition, ABCC4 facilitates the transport of sphingosine-1-phosphate (S1P), the main active metabolite of sphingolipids, from the platelets [14].

ABCC4 is ubiquitously expressed, with particularly high expression in hematopoietic stem cells and blood cells [15]. Limited data on the functions of its variants are available despite the fact that *ABCC4* is a highly polymorphic gene. *ABCC4* variants are associated with various diseases; however, no report has implicated an association between *ABCC4* and immunological diseases. Copsel et al. demonstrated that an *ABCC4* polymorphism regulates the cellular levels of cAMP and controls human leukemia cell proliferation and differentiation, indicating its role in cellular processes [16]. Therefore, *ABCC4* variants may also play an important role in the pathogenesis of asthma.

There is little evidence regarding associations of *ABCC4* polymorphisms with asthma in Korean patients. Therefore, we investigated the potential associations between asthma and *ABCC4* polymorphisms in a Korean population.

## 2. Materials and Methods

### 2.1. Study Subjects

We enrolled 270 asthma patients and 120 normal healthy controls (NCs) from the Department of Allergy and Clinical Immunology, Ajou University Hospital, Suwon, Korea. Written informed consent was obtained from each subject, and the study was approved by the Institutional Review Board of Ajou University Hospital (AJIRB-GEN-SMP-13-108).

Methacholine bronchial challenge tests were performed as described previously [17]. NCs were selected from the general population using a screening questionnaire. Participants with a history of respiratory symptoms or aspirin hypersensitivity were excluded. All NC subjects exhibited a forced expiratory volume 1 (FEV_1_) > 80% of the predicted value, a provocation concentration (PC_20_) of methacholine > 25 mg/mL and normal findings on chest radiographs. Atopy was defined as one or more positive reactions on a skin prick test of 12 common aeroallergens (Bencard Co., Brendford, UK); histamine and saline served as controls. Serum total IgE levels were measured using the UniCAP system (Thermo Scientific, Uppsala, Sweden) according to the manufacturer's instructions. The threshold cut-off value for a specific IgE level was 0.35 kU/L, as measured by UniCAP. The presence of rhinosinusitis and nasal polyps was determined using paranasal sinus X-ray and rhinoscopy.

### 2.2. DNA Extraction, Single Nucleotide Polymorphism (SNP) Identification, and Genotyping

Each of the twenty Korean asthma patients and NCs was used for SNP identification. Total genomic DNA was isolated from peripheral blood samples using the Puregene DNA Purification Kit (Gentra, Minneapolis, MN, USA) according to the manufacturer's protocol. Our objective was to screen for promoter and 5′-untranslated region (UTR) SNPs. Based on previous findings and sequencing results, we chose two SNPs: one in the promoter and one in the 5′UTR of *ABCC4* (−1508A>G and −642C>G, resp.). The two SNPs were genotyped using the TaqMan Allelic Discrimination assay with TaqMan probes (rs868853, −1508A>G assay ID c__7461591_10; rs869951, −642C>G, c__7461587_10; Applied Biosystems, Foster City, CA, USA).

### 2.3. Quantification of Serum and Urinary Metabolites

For the serum and urinary metabolites, we enrolled 60 and 31 asthmatic patients for measurement, respectively. The serum and urinary levels of S1P and five eicosanoid metabolites, LTE4, prostaglandin F2*α* (PGF2*α*), thromboxane B2 (TXB2), 15-hydroxyeicosatetraenoic acid (15-HETE), and eoxin C4, were determined using the Agilent 6530 quadrupole time-of-flight (Q-TOF) mass spectrometer. The device settings have been described in detail in a previous study [18].

### 2.4. Measurement of Serum Inflammatory Cytokines

Several inflammatory biomarkers, including myeloperoxidase (MPO), interleukin- (IL-) 8, IL-18, eotaxin-1, and eotaxin-2, were measured by enzyme-linked immunosorbent assay (ELISA) (Quantikine, R&D Systems, Minneapolis, MN, USA). Serum periostin levels were measured using a proprietary sandwich ELISA kit (Shino-test, Kanagawa, Japan) [19]. Each sample was run in duplicate. Serum samples were stored at −80°C prior to use.

### 2.5. Activity of the *ABCC4* Promoter Constructs

Human mast cells (HMC-1) were cultured in Iscove's Modified Dulbecco's Medium (Gibco, Grand Island, NY, USA). A549, human alveolar type II epithelial-like, and U937, human leukemic monocyte lymphoma cell lines, were cultured in Roswell Park Memorial Institute-1640 Medium (Gibco) supplemented with 10% heat-inactivated fetal bovine serum, 100 U/mL penicillin G sodium, and 100 *μ*g/mL streptomycin sulfate (Gibco) at 37°C in a 5% CO_2_ incubator.

A 1681 bp fragment of the human *ABCC4* gene was amplified from the genomic DNA of −1508GG and −1508AA homozygous subjects by PCR using the following primers: (forward) 5′-TCTATCGATAGGTACGGCCATGCTTAGACATAGGCTTA-3′ and (reverse) 5′-GATCGCAGATCTCGAAGAACACGCGTGAGCAGAGGTT-3′. PCR products were gel purified using an Agarose Gel Purification Kit (GeneAll Biotechnology, Seoul, Korea) and ligated into the pGL3-basic vector (Promega, Madison, WI, USA) after digestion with KpnІ and XhoІ (Takara, Shuzo, Japan) using the In-Fusion^®^ HD Cloning Kit (Clontech Laboratories Inc., Mountain View, CA, USA). All constructs were confirmed by a restriction enzyme analysis and DNA sequencing. Plasmid DNAs were prepared from these constructs using the Endo-Free Plasmid Maxi Kit (Qiagen, Hilden, Germany), and the concentration and purity were assessed by UV spectrophotometry and agarose gel electrophoresis. Before transfection, the constructs were verified by direct sequencing.

The constructs were transfected into A549, HMC-1, and U937 cells using Lipofectamine (Invitrogen) according to the manufacturer's protocol. Briefly, 1 × 10^5^ cells were seeded into 12-well plates and, after reaching 70–80% confluency, were transfected with 1 *μ*g of the reporter construct, 5 *η*g Renilla plasmid DNA and 5 *μ*L Lipofectamine. Forty-eight hours after transfection, the cells were lysed and assayed for firefly luciferase activity according to the manufacturer's instructions (Promega). Transfection and luciferase assays were repeated three times according to the method described above.

### 2.6. Statistical Analyses

Statistical analyses were performed using SPSS version 22.0 (SPSS Inc., Chicago, IL, USA). Differences in clinical characteristics among the groups were examined using the independent *t*-test for continuous variables and the *χ*^2^ test for categorical variables. Genotype frequency was examined between the subject groups using a *χ*^2^ test, and differences in clinical characteristics, cytokines and metabolites, according to genotype were examined using a logistic regression analysis with codominant, dominant, and recessive models after accounting for age and sex as covariables. Statistical significance was established at *P* < 0.05.

## 3. Results

### 3.1. Clinical Characteristics of the Study Subjects

The clinical characteristics of the study population are summarized in Table 1. The mean age of the asthma patients was 43.8 (±13.85) years and that of the NCs was 27.04 (±7.17) years (*P* < 0.001). The percentage of males was significantly higher in the asthma group (58.21%) than in the NC group (38.35%) (*P* < 0.001). In the asthma group, atopy was observed in 48.50% of patients, rhinosinusitis in 83.75%, and nasal polyps in 39.44%.

### 3.2. No Association of the *ABCC4* Promoter Polymorphisms with Asthma

Two promoter polymorphisms of *ABCC4* gene (−1508A>G and −642C>G) were examined in this study. Linkage disequilibrium analysis was performed between the two *ABCC4* SNPs. Three common haplotypes, ht1 [AC], ht2 [AG], and ht3 [GG], were constructed using the EM algorithm (Table 2), which revealed the genotype and haplotype frequencies of SNPs in the study subjects. There were no significant differences with respect to genotype or haplotype between the study groups.

### 3.3. Associations between the *ABCC4* −1508A>G Polymorphism and Urinary Levels of Metabolites

We next examined potential associations between *ABCC4* gene polymorphisms and serum and urinary eicosanoid metabolites and S1P (Table 3).

**Figure 1:**
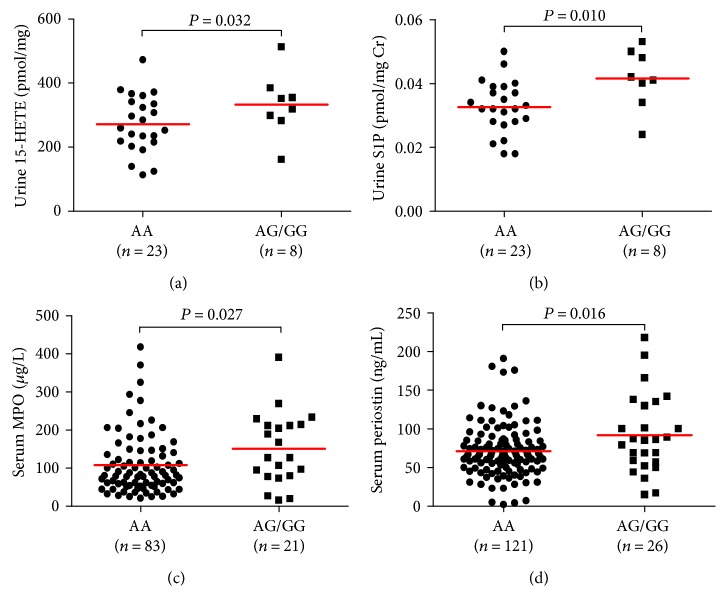
Asssociation of the baseline levels of (a) urinary 15-HETE, (b) urinary S1P, (c) serum MPO, and (d) serum periostin with the *ABCC4* −1508A>G polymorphisms in asthma patients. *ABCC4*: ATP-binding cassette subfamily C member 4; 15-HETE: 15-hydroxyeicosatetraenoic acid; MPO: myeloperoxidase; S1P: sphingosine-1-phosphate. *P* values were obtained by logistic regression with age and sex as covariates. The data represented as mean values ± SD.

Among five eicosanoid metabolites (LTE4, PGF2*α*, TXB2, 15-HETE, and eoxin C4), the urinary 15-HETE level was significantly associated with the *ABCC4* −1508A>G polymorphism; asthma patients carrying the −1508G allele showed a significantly higher level than that of noncarriers (332 ± 99.31 versus 271.91 ± 89.87 pmol/mg creatinine [pmol/mg Cr], *P* = 0.032; Figure 1(a)). Serum levels of LTE4, PGF2*α*, TXB_2,_ and eoxin C4 did not differ significantly between −1508G carriers and noncarriers among asthma patients (Table 3).

Regarding to S1P, asthma patients carrying the −1508G allele showed a significantly higher level of the urinary level of S1P than that of noncarriers (41.5 ± 9.35 versus 32.56 ± 8.25 pmol/mg Cr, *P* = 0.010; Figure 1(b)).

### 3.4. Associations between the *ABCC4* −1508A>G Polymorphism and Clinical Characteristics, Serum Periostin and MPO

Several inflammatory cytokines that are important biomarkers for asthma were also measured (Table 3). Among the cytokines examined, asthma patients carrying the −1508G allele showed significantly higher serum MPO levels (150.91 ± 94.13 versus 108.26 ± 79.5 mg/L, *P* = 0.027) and serum periostin level than those of noncarrier asthma patients (91.83 ± 50.85 versus 71.07 ± 33.62 ng/mL, *P* = 0.016), (Figures 1(c) and 1(d)).

### 3.5. Effects of the *ABCC4* Polymorphisms on Transcriptional Activity

**Figure 2:**
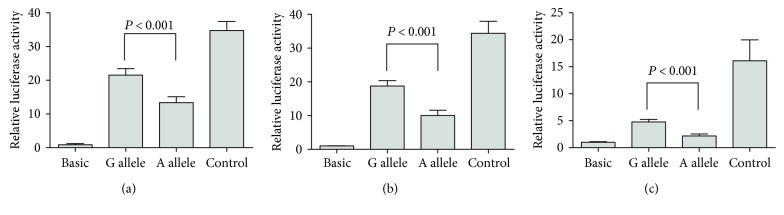
Effect of the *ABCC4* −1508A>G polymorphism on the promoter activity. Transfection of plasmid constructs carrying *ABCC4* −1508A or G allele into (a) A549, (b) U937, and (c) HMC-1 cells. All *P* values were obtained by Mann–Whitney *U* test. Data represent the mean values of three independent experiments ± SD. Each experiment was conducted in triplicate.

The luciferase reporter assay was performed using constructs containing two different *ABCC4* alleles, −1508A and −1508G, to determine the transcriptional effects of the *ABCC4* –1508A>G polymorphism. The constructs comprised of the *ABCC4* sequence and a luciferase reporter gene were transfected into A549, U937, and HMC-1 cells. The reporter activities of the −1508G allele and −1508A allele constructs were compared. Luciferase activity was significantly enhanced in the construct with the −1508G allele compared with the −1508A allele in all cell lines (*P* < 0.01 for each, Figure 2).

## 4. Discussion

Asthma is a complex, chronic respiratory disease with a wide clinical spectrum, with contributions from several environmental and genetic factors [20, 21]. Recent studies have shown that various gene polymorphisms influence the onset and progression of asthma [22, 23]. In the present study, we selected two SNPs within the *ABCC4* gene to examine their potential roles in asthma pathogenesis based on their relationships with eicosanoid, sphingolipid metabolites, and proinflammatory cytokines. To date, the roles of these genes in asthma have not been determined.

Most studies on ABCC4 have focused on its role in cancer chemotherapy, particularly its ability to confer clinical drug resistance [24]. Diverse studies have shown that ABCC4 induces the extrusion of cyclic nucleotides in various cell types; however, it has emerged as the main transporter of cAMP [25]. van de Ven et al. reported that ABCC4 plays an important role in dendritic cell migration in humans and that inhibition of ABCC4 activity decreases dendritic cell migration in the skin [13]. However, no association study to date has examined the relationships between this gene and immunological diseases such as asthma, although this gene is associated with immunological processes.

We first identified a significant association between the *ABCC4* −1508A>G polymorphism and the urinary levels of metabolites, including 15-HETE and S1P. 15-HETE is the major metabolite of arachidonic acid in the 15-lipoxygenase pathway [26]. 15-HETE was recently proposed as a biomarker for asthma severity, as its levels were 5-fold higher in eosinophils from severe asthmatics than from mild asthmatics [27]. 15-HETE undergoes reaction to produce 14,15-epoxides, designated eoxins A4, C4, D4, and E4 in eosinophils, mast cells, and nasal polyps from allergic subjects [28]. Similar to cysteinyl leukotrienes, eoxins are potent proinflammatory agents [28]. We found a tendency toward increased eoxin C4 levels in asthmatics with the −1508G allele, although no significance was observed, suggesting inflammation of the airways in eosinophilic asthma. Beside, S1P, a major metabolite of sphingolipid pathway, has been identified as a biomarker for asthma in our previous study [29]. S1P is suggested to contribute to airway hyperreactivity and release of IL-4 and IL-13, thereby involving in asthma pathogenesis [30, 31]. Inhibitors of ABCC4 block the release of S1P from platelet granules [14]. Therefore, the −1508G allele of *ABCC4* polymorphism may be associated with the transport of metabolites from immune cells.

Secondly, we found enhanced serum periostin levels in asthma patients carrying the −1508G allele [32]. Periostin is a multifunctional protein expressed in many types of inflammatory cells, such as epithelial cells, mast cells, and so forth [33]. Recent studies have suggested that periostin modulates Th2-mediated asthma pathogenesis by assisting in the recruitment of inflammatory cells, particularly eosinophils, to the lungs [34]. Moreover, periostin is known to facilitate the activation of dendritic cells, thereby rendering airway hyperresponsiveness and airway inflammation in mice [35, 36]. Beside, we discovered an increased serum level of MPO in the *ABCC4* −1508G allele carriers comparing to that of noncarriers. Activated neutrophils secrete MPO which then induces oxidative stress, thereby resulting in oxidative damage of respiratory cells, lung inflammation, cytotoxicity, airway obstruction, and decrease of lung function [37, 38]. A polymorphism of the *MPO* gene was proposed to be associated with asthma susceptibility [38]. Taken together, the *ABCC4* –1508G allele may also interfere the release of periostin and MPO from inflammatory cells.

Although we did not find any genetic association in asthma patients in the present study, we found that a functional polymorphism of the *ABCC4* gene (−1508A>G) may affect its promoter activity, thereby affecting release of 15-HETE, S1P, periostin, and MPO from innate immune cells in asthma.

## 5. Conclusions

To our knowledge, this is the first study to provide evidence of associations between *ABCC4* and 15-HETE, S1P, periostin, and MPO in asthma patients. The present findings further suggest that ABCC4 represents a new potential target of asthma therapy. However, further studies are required to understand the functional mechanism of *ABCC4* polymorphisms on airway inflammation in asthmatics.

## Figures and Tables

**Table 1:** Clinical characteristics of study subjects.

	Asthmatics (*n* = 270)	NC (*n* = 120)	*P* value
Age (years)^†^	43.80 ± 13.85	27.04 ± 7.17/98	**<0.001**
Male (%)^‡^	58.21	38.35	**<0.001**
Atopy (%)^†^	48.50	NA	NA
Total IgE (kU/L)^†^	0.431 ± 0.9	NA	NA
Rhinosinusitis (%)^‡^	83.75	NA	NA
Nasal polyp (%)^‡^	39.44	NA	NA
FEV_1_ (%)^†^	85.69 ± 20.30	NA	NA
PC_20_ methacholine (mg/mL)	9.17 ± 15.99	NA	NA
Asthma duration (years)^†^	7.69 ± 15.97	NA	NA

NC: normal control; *n*: number of subjects; NA: not applicable; FEV_1_: forced expiratory volume in 1 s; PC_20_ methacholine: provocative concentration of methacholine producing a 20% fall in FEV_1_. ^**†**^This value was presented as mean ± SD, whereas ^‡^ value was shown as percentage. Values in bold indicate significant *P* value. Each *P* value <0.05 was considered to be significant. *P* value was obtained by *t*-test for continuous variables and the *χ*^2^ test for categorical variables.

**Table 2:** Distribution of genotypes and haplotypes of *ABCC4* gene polymorphisms.

SNP	Genotype	Asthmatics (*n* = 270)	NC (*n* = 120)	*P* value
−1508A>G (rs868853)	AA	224 (83.3)	102 (86.4)	0.288
	AG	47 (17.5)	16 (13.6)	0.417
	GG	2 (0.74)	2 (1.69)	0.648
−642C>G (rs869951)	CC	94 (34.9)	52 (44.1)	0.61
	CG	139 (51.7)	47 (39.8)	0.101
	GG	36 (13.4)	19 (16.1)	0.068
ht1 [AC]	**+/+**	94 (34.9)	52 (44.0)	0.171
	**+/−**	139 (51.7)	47 (39.8)	0.705
	**−/−**	36 (13.4)	19 (16.1)	0.068
ht2 [AG]	**+/+**	17 (6.3)	14 (11.9)	0.077
	**+/−**	156 (57.9)	50 (42.3)	0.094
	**−/−**	96 (35.7)	54 (45.7)	0.366
ht3 [GG]	**+/+**	2 (0.74)	2 (1.7)	0.404
	**+/−**	17 (6.3)	3 (2.5)	0.75
	**−/−**	250 (92.9)	113 (95.7)	0.454

NC: normal healthy control; *n*: number of subjects; ht: haplotype. *P* value was obtained by logistic regression analysis with age and sex as covariates.

**Table 3:** Association of *ABCC4* −1508A>G polymorphism with clinical features, metabolite, and cytokine profiles in asthmatic patients.

Clinical features	AA (*n* = 305)	AG/GG (*n* = 48)	*P* value

Age (years)^†^	39.01 ± 14.34	40.92 ± 15.09	0.735
Sex, male (%)^‡^	32.14	28.57	0.335
Atopy (%)^‡^	57.46	51.18	0.928
Total IgE (IU/mL)^†^	451.48 ± 965.78	278.32 ± 319.68	0.240
Rhinosinusitis (%)^‡^	81.21	18.78	0.143
Nasal polyp (%)^‡^	81.11	18.88	0.889
FEV_1_ (%)^†^	86.45 ± 19.66	87.54 ± 14.57	0.638
PC_20_ methacholine (mg/mL)	10.19 ± 17.07	5.82 ± 8.04	0.119
Total IgE	451.48 ± 965.78	278.32 ± 319.69	0.24
Asthma duration (years)^†^	6.30 ± 6.03	12.81 ± 32.36	0.237

Inflammatory cytokines	AA (*n* = 121)	AG/GG (*n* = 26)	*P* value

MPO (*μ*g/L)^†^	108.26 ± 79.5	150.91 ± 94.13	**0.027**
IL-8 (pg/mL)^†^	16.82 ± 14.45	14.47 ± 7.5	0.616
IL-18 (pg/mL)^†^	247.77 ± 166.06	281.92 ± 197.10	0.560
Eotaxin-1 (ng/mL)^†^	85.67 ± 63.67	85.22 ± 65.11	0.841
Eotaxin-2 (ng/mL)^†^	1146.92 ± 802.14	1104.95 ± 641.09	0.650
Periostin (ng/mL)^†^	71.07 ± 33.62	91.83 ± 50.85	**0.016**

Serum metabolites (ng/mL)	AA (*n* = 43)	AG/GG (*n* = 17)	*P* value

15-HETE	265.98 ± 185.69	334.34 ± 132.47	0.195
LTE4	18.22 ± 17	18.8 ± 14.71	0.731
PGF2*α*	23.55 ± 9.3	21.96 ± 8.69	0.653
TXB2	0.41 ± 0.42	0.39 ± 0.17	0.952
Eoxin C4	4.82 ± 9.5	8.14 ± 11.95	0.224
S1P	111.75 ± 42.16	120.91 ± 42.88	0.419

Urinary metabolites (pmol/mg Cr)	AA (*n* = 23)	AG/GG (*n* = 8)	*P* value

15- HETE	271.91 ± 89.87	332.5 ± 99.31	**0.032**
LTE4	7648.29 ± 14378.31	2601.73 ± 3043.59	0.616
PGF2*α*	8618.51 ± 22942.8	1678.71 ± 978.76	0.65
TXB2	5686.61 ± 4305.7	4798.25 ± 3957.21	0.765
Eoxin C4	144.78 ± 94.07	124.75 ± 46.65	0.602
S1P	32.56 ± 8.25	41.5 ± 9.35	**0.010**

^†^Values were presented as mean ± SD, whereas ^‡^ values were shown as percentage. Cr: creatinine; LTE4: leukotriene E4; PGF2*α*: prostaglandin F2*α*; TXB2: thromboxane B2; 15-HETE: 15-hydroxyeicosatetraenoic acid; MPO: myeloperoxidase; S1P: sphingosine-1-phosphate. *P* value was obtained by logistic regression analysis with age and sex as covariates.
